# A Prospective, Observational, Multicentre Study Concerning Nontechnical Skills in Robot-assisted Radical Cystectomy Versus Open Radical Cystectomy

**DOI:** 10.1016/j.euros.2020.05.003

**Published:** 2020-07-03

**Authors:** Alexander J.W. Beulens, Willem M. Brinkman, Evert L. Koldewijn, Ad J.M. Hendrikx, Jean Paul A. van Basten, Jeroen J.G. van Merriënboer, Henk G. Van der Poel, Chris H. Bangma, Cordula Wagner

**Affiliations:** a Netherlands Institute for Health Services Research (NIVEL), Utrecht, The Netherlands; b Department of Urology, Catharina Hospital, Eindhoven, The Netherlands; c Department of Oncological Urology, University Medical Centre Utrecht, Utrecht, The Netherlands; d Department of Urology, Canisius Wilhelmina Hospital, Nijmegen, The Netherlands; e School of Health Professions Education, Maastricht University, Maastricht, The Netherlands; f Department of Urology, Netherlands Cancer Institute-Antoni van Leeuwenhoek Hospital, Amsterdam, The Netherlands; g Department of Urology, Erasmus University Medical Centre, Rotterdam, The Netherlands; h Amsterdam Public Health Research Institute, Amsterdam UMC, Location VUmc, Amsterdam, The Netherlands

**Keywords:** Nontechnical skills, Cystectomy, Surgical skills, Outcome, Robot-assisted surgery

## Abstract

**Introduction and hypotheses:**

valuation of surgical skills, both technical and nontechnical, is possible through observations and video analysis. Besides technical failures, adverse outcomes in surgery can also be related to hampered communication, moderate teamwork, lack of leadership, and loss of situational awareness. Even though some surgeons are convinced about nontechnical skills being an important part of their professionalisation, there is paucity of data about a possible relationship between nontechnical skills and surgical outcome. In robot-assisted surgery, the surgeon sits behind the console and is at a remote position from the surgical field and team, making communication more important than in open surgery and conventional laparoscopy. A lack of structured research makes it difficult to assess the value of the different analysis methods for nontechnical skills, particularly in robot-assisted surgery. Our hypothesis includes the following: (1) introduction of robot-assisted surgery leads to an initial decay in nontechnical skills behaviour during the learning curve of the team, (2) nontechnical skills behaviour is more explicitly expressed in experienced robot-assisted surgery teams than in experienced open surgery teams, and (3) introduction of robot-assisted surgery leads to the development of different forms of nontechnical skills behaviour compared with open surgery.

**Design:**

This study is a prospective, observational, multicentre, nonrandomised, case-control study including bladder cancer patients undergoing either an open radical cystectomy or a robot-assisted radical cystectomy at the Catharina Hospital Eindhoven, the Netherlands, or at the Netherlands Cancer Institute, Antoni van Leeuwenhoek Hospital Amsterdam. All patients are eligible for inclusion; there are no exclusion criteria. The Catharina Hospital Eindhoven, the Netherlands, performs on average 35 radical cystectomies a year. The Netherlands Cancer Institute, Antoni van Leeuwenhoek Hospital Amsterdam, performs on average 100 radical cystectomies a year.

**Protocol overview:**

The choice of treatment is at the discretion of the patient and the surgeon. Patient results will be obtained prospectively. Pathology results as well as complications occurring within 90 d following surgery will be registered. Surgical complications will be registered according to the Clavien-Dindo system.

**Measurements:**

Nontechnical skills will be observed using five different methods: (1) NOTSS: Nontechnical Skills for Surgeons; (2) Oxford NOTECHS II: a modified theatre team nontechnical skills scoring system; (3) OTAS: Observational Teamwork Assessment for Surgery; (4) Interpersonal and Cognitive Assessment for Robotic Surgery (ICARS): evaluation of nontechnical skills in robotic surgery; and (5) analysis of human factors. Technical skills in robot-assisted radical cystectomy will be analysed using two different methods: (1) GEARS: Global Evaluative Assessment of Robotic Skill and (2) GERT: Generic Error Rating Tool.

**Safety criteria and reporting:**

Formal ethical approval has been provided by Medical research Ethics Committees United (MEC-U), The Netherlands (reference number W19.048). We hope to present the results of this study to the scientific community at conferences and in peer-reviewed journals.

**Statistical analysis:**

Frequency statistics will be calculated for patient demographical data, and a Shapiro-Wilk test with *p* > 0.05 will be used to define normal distribution. Univariate analysis will be conducted to test for statistically significant differences in observation scores between open radical cystectomy and robot-assisted radical cystectomy cohorts across all variables, using independent sample *t* tests and Mann-Whitney *U* testing, as appropriate. A variable-selection strategy will be used to create multivariate models. Binary logistic regression will be conducted to calculate odds ratios and 95% confidence intervals for significant predictors on univariate analysis and clinically relevant covariates. Statistical significance is set at *p* < 0.05 based on a two-tailed comparison.

**Summary:**

This study uses a structured approach to the analysis of nontechnical skills using extracorporeal videos of both open radical cystectomy and robot-assisted radical cystectomy surgeries, in order to obtain detailed data on nontechnical skills during open and minimally invasive surgeries. The results of this study could possibly be used to develop team-training programmes, specifically for the introduction of the surgical robot in relation to changes in nontechnical skills. Additional analysis of technical skills using the intracorporeal footage of the surgical robot will be used to elucidate the role of surgical skills and surgical events in nontechnical skills.

## .

This study uses a structured approach to the analysis of nontechnical skills (NTS) using extracorporeal videos of both open radical cystectomy and robot-assisted radical cystectomy surgeries, in order to obtain detailed data on NTS during open and minimally invasive surgery.

## Introduction and hypotheses

Qualification and certification of the performance of surgical skills are still in a preliminary phase within all surgical specialties, including urology. There are, however, urgent calls from the government and patient organisations for well-defined proficiency standards to safeguard the quality of care [1], [2]. In addition, professionals themselves are increasingly interested to define their qualifications and improve skills [3].

Multiple research groups are investigating the relation between surgeons’ technical skills and postoperative outcome [4], [5], [6]. With the introduction of laparoscopy and the surgical robot, new and improved assessment tools of surgical skills have been developed [5], [7], [8], [9].

Although the analysis of technical surgical skills in robot-assisted surgery can lead to major improvements of postoperative outcomes [10], the possible influence of nontechnical skills on postoperative outcomes also merits attention.

The nontechnical skills needed for a successful robot-assisted radical cystectomy probably differ from those needed for an open radical cystectomy.

Even though several general assessment methods have been developed for both the entire team [11], [12], [13] and individual team members [14], [15], [16], the question remains whether these tools can accurately assess nontechnical skills in complex robot-assisted surgeries such as robot-assisted radical cystectomy. With the introduction of the Interpersonal and Cognitive Assessment for Robotic Surgery (ICARS) [17], adaptation to the robot-assisted surgical setting has started.

The introduction of the surgical robot has totally changed the traditional set-up of the operating room (OR), since the scrub nurse and the surgeon are no longer on opposite sides of the patient. In robot-assisted surgery, the surgeon is located in a separate control console during most of the surgery, and therefore direct communication with team members could be hampered. It is conceivable that loss of nonverbal communication can influence the workflow and therefore the quality of the performance, including patient's safety.

Two systematic reviews concerning the studies of nontechnical skills in minimally invasive surgery (ie, conventional laparoscopy and robot-assisted surgery) have been published [18], [19]. A wide variety of tools were used in assessments of nontechnical skills, which makes comparison of tools difficult [18], [19].

van der Vliet et al [19] advises additional research in nontechnical skills to be performed in different surgical approaches (open, laparoscopic, and robot assisted). Moreover, the use of multiple trained observers is advised for assessing audiovisual recordings of the surgical environment to identify and quantify possible interobserver reliability. The group of Gjeraa et al [18] advises systematic identification of nontechnical skills in minimally invasive surgery in order to develop effective, evidence-based team training programmes for minimally invasive surgeries.

The present study aims to perform a structured evaluation of nontechnical skills in both open and robot-assisted complex surgery, to investigate the manner in which the introduction of a surgical robot influences both nontechnical skills and surgical outcomes during the 1st year of robot-assisted radical cystectomy compared with open radical cystectomy.

In addition, analysis of technical skills in robot-assisted radical cystectomy will be performed to evaluate the possible relationship between technical and nontechnical skills. Radical cystectomy was chosen for this analysis because it is a lengthy, complex, and demanding surgery for the surgeon and other team members.

Since radical cystectomy surgeries take many hours to complete, a long-term and detailed analysis is possible per procedure. Radical cystectomy is traditionally performed using an open surgical approach (open radical cystectomy) at the Catharina Hospital Eindhoven, but recently a shift is made to robot-assisted radical cystectomy. This shift enables us to investigate in which manner nontechnical skills change during the introduction of robot-assisted radical cystectomy. The nontechnical skills during the learning curve of robot-assisted radical cystectomy in the Catharina Hospital Eindhoven will be compared with the nontechnical skills during the open radical cystectomy in the same hospital as well the nontechnical skills of an experienced robot-assisted radical cystectomy team in the Antoni van Leeuwenhoek Hospital.

These analyses will be performed in order to investigate in which manner nontechnical skills change during the introduction of the robot-assisted radical cystectomy and which factors contribute to the learning curve. Results obtained during this study could be beyond robot-assisted radical cystectomy, since the changes in operating OR set-up and the loss of nonverbal communication are universal when making the shift from open to robot-assisted surgery.

Our hypothesis includes the following:

1. Introduction of robot-assisted surgery leads to an initial decay in nontechnical skills behaviour during the learning curve of the team.
2. In experienced robot-assisted surgery teams, nontechnical skills behaviour is more explicitly expressed than in experienced open surgery teams.
3. Introduction of robot-assisted surgery leads to the development of different forms of nontechnical skills behaviour compared with open surgery.

The results of this study could possibly be used to develop team training programmes specifically for the introduction of the surgical robot in relation to changes in nontechnical skills. Additional analysis of technical skills using the intracorporeal footage of the surgical robot will be used to elucidate the role of surgical skills and surgical events in nontechnical skills.

## Design

The present study is a prospective, observational, multicentre, nonrandomised, case-control study that will include all patients undergoing either an open radical cystectomy or a robot-assisted radical cystectomy in Catharina Hospital Eindhoven or Antoni van Leeuwenhoek Hospital.

### Time scales

**Fig. 1:**
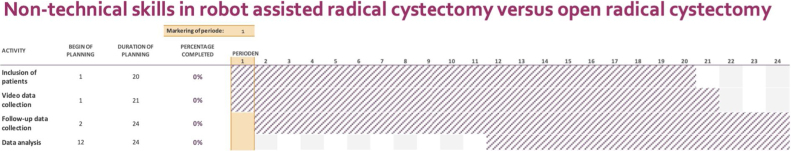
Timeline of the study.

The inclusion will be from January 2021 until August 2022 in both hospitals simultaneously (Fig. 1). Video collection will start once the first patient is included and will continue until the last patient has had their surgery. Follow-up data collection will start in February 2021 and will continue until December 2022. Data analysis will start in January 2022.

### Study population

The surgical team in the OR will be the study population. Individual permissions will be obtained from all members of the surgical team, that is, urologists, OR nurses, and anaesthesiologists. Surgeries will be performed by three urologists, one surgeon will perform all open radical cystectomies, one surgeon will perform all robot-assisted radical cystectomies in the Catharina Hospital Eindhoven, and one surgeon will perform all robot-assisted radical cystectomies and open radical cystectomies in the Antoni van Leeuwenhoek Hospital. OR nurses for each open radical cystectomy and robot-assisted radical cystectomy in the Catharina Hospital Eindhoven will be selected based on shift schedules from the experienced dedicated team of six urology OR nurses. OR nurses for each surgery in the Antoni van Leeuwenhoek Hospital will be selected from the experienced dedicated team of six urology OR nurses. Anaesthesiologists will be selected randomly for each surgery from the total number of anaesthesiologists who have signed an informed consent form. All team members have worked together before.

After five robot-assisted radical cystectomy procedures, a survey based on the survey developed by McBride et al [20] (Supplementary material) will be held among the OR nurses in the Catharina Hospital Eindhoven in order to investigate the point of view of the OR nurses on the potential benefits of robot-assisted surgery. All surgeons will be asked what level of prior experience/training they have prior to the start of the study.

### Inclusion criteria

Patients who will undergo either an open radical cystectomy or a robot-assisted radical cystectomy in Catharina Hospital Eindhoven or Antoni van Leeuwenhoek Hospital are eligible for this study. The choice of treatment is at the discretion of the patient and the surgeon.

For study inclusion, the following criteria must be met:

1. Patients must be aged ≥18 yr.
2. Patients must be able to understand and sign an informed consent form.
3. Patients will undergo either an open radical cystectomy or a robot-assisted radical cystectomy in Catharina Hospital Eindhoven or Antoni van Leeuwenhoek hospital.
4. Indication for the radical cystectomy must be urothelial cell carcinoma of the bladder.
5. Informed consent of the patient must be obtained to gather data and perform observations during surgery.

### Exclusion criteria

No exclusion criteria will be used for this study.

### Recruitment and consent

Informed consent from both patient and OR staff will be obtained, allowing observation of the surgical procedure and collection of patient data.

### Withdrawal of individuals/employees

Both the patient to undergo the surgery and all employees present during the surgery can always withdraw their consent to the use of their personal data/recording of the surgery. The data collected up to the moment of withdrawal of consent and the recording of the surgery will be destroyed after consent has been withdrawn. Consent can be withdrawn up to 6 mo after surgery in order to have the recorded surgery destroyed. After 6 mo, the recorded surgery will be automatically destroyed.

### Centre details

Based on prior data, on average a total of 35 open radical cystectomies are performed yearly in the Catharina Hospital Eindhoven. Since the robot-assisted radical cystectomy has just been introduced, the total number of open radical cystectomies will be divided over the open radical cystectomy and the robot-assisted radical cystectomy modalities; it is expected that half of the radical cystectomies will be performed using a robot–assisted surgical approach. In the Antoni van Leeuwenhoek Hospital, on average a total of 50 robot-assisted radical cystectomies and 50 open radical cystectomies are performed each year. It is possible to include further hospitals in the future.

## Protocol overview

Patient results will be obtained prospectively. Pathology results will be registered as well as complications occurring within 90 d following surgery. Complications will be registered according to the Clavien-Dindo system surgery.

### Measurements

Nontechnical skills will be observed using five different methods:

1. NOTSS: Nontechnical Skills for Surgeons [16].
2. Oxford NOTECHS II: a modified theatre team nontechnical skills scoring system [11], [12].
3. OTAS: Observational Teamwork Assessment for Surgery [21].
4. ICARS: evaluation of nontechnical skills in robotic surgery [17].
5. Analysis of human factors [22].

No intracorporeal videos of open radical cystectomy can be recorded due to blocking of the image by the surgeons and the OR lights, and difficulty getting a clear view into the surgical area in the pelvic region from a distance. The analysis of technical skills will be performed only using the robot-assisted radical prostatectomy videos. This method of analysis will be performed to investigate the influence of robot-assisted surgery experience on nontechnical skills and outcome of the surgery. Technical skills in robot-assisted radical cystectomy will be analysed using two different methods:

1. GEARS: Global Evaluative Assessment of Robotic Skill [7].
2. GERT: Generic Error Rating Tool [5].

### Data collection and handling

Data collection will consist of video capturing and analysis of patient records. Two trained observers (observer 1 and 2, both have a background in medicine), with orientation and training in both nontechnical skills and technical assessment methods, will independently observe surgical videos. All videos will be analysed by both researchers. In case of disagreement, a third independent expert with a psychology and leadership assessment background (observer 3) will be asked to perform a third analysis. Inter-rater reliability will be analysed using Cohen's kappa.

**Fig. 2:**
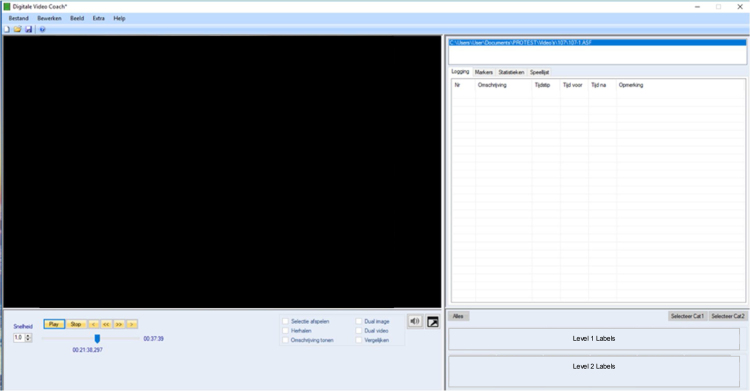
Overview of the video analysis software Digital Video Coach.

The surgical video will be assessed in multiple phases; in each phase, a nontechnical skills assessment method will be used to assess nontechnical skills. Surgical videos will be analysed using customisable video analysis software “Digital Video Coach” developed by ZEAL IT, Einhoven, The Netherlands (Fig. 2).

The video analysis software “Digital Video Coach” makes it possible to register the occurrence of nontechnical skills behaviour and perioperative events (ie, people entering or leaving the OR, phone calls, etc.) Two sets of labels will be created in order to define the different nontechnical skills behaviour and perioperative events present during the surgery. The selection of one of the labels automatically marks the time code corresponding to the moment the label was pressed. This makes it possible to measure the duration of the nontechnical skills behaviour and perioperative events. The labels used for this analysis will be specific to the assessment method of nontechnical skills.

Training of the two observers (observers 1 and 2) will be performed using the NOTSS introductory course and advanced course (NOTSS for Trainees and NOTSS in a Box) as developed by the Royal College of Surgeons of Edinburgh [23]. Further training in the remaining analysis methods will be provided by a specialist in the assessment of nontechnical skills (observer 3).

The technical skills assessment training will be provided by an expert in technical skills analysis with proficient knowledge of the procedure and robot-assisted surgery (observer 4, who is a surgeon who has performed over 200 open radical cystectomy and robot-assisted radical cystectomy procedures and is a trainer of new and experienced surgeons). Observer 4 will act as independent expert in case of disagreement between the two observers (observers 1 and 2).

The videos will be recorded using three cameras installed at three different points in the OR. Objects that should be in view are the OR table, robot console in case of robot-assisted surgery, anaesthesiology equipment, OR door to the nonsterile area of the OR complex, and OR door to the sterile area of the OR. Recording from three different angles in the OR will ensure that there will be a 360° view of the proceedings in the OR. The cameras used have a 170° image angle with high-definition imaging so that maximum coverage can be achieved.

Voice data will be collected using personalised voice recorders per staff member present in the OR. The audio feed on the cameras is strong enough to get a general view of the conversations during the surgery; for detailed analysis, recording of the personal voice recorder will be used to gain insight into the orders given during difficult or abnormal phases of the surgery.

Surgeon-specific data will be recorded at the start of the operation, which include but are not limited to the age, gender, right or left handedness, gaming experience, and prior surgical and robot-assisted surgery experience of the surgeon. If multiple surgeons participate in the same surgery, all will be asked to complete the above-mentioned questionnaire; changes in lead surgeon will be recorded during the surgery.

Cases will be deidentified and labelled with study codes. Patient data will be recorded during regular follow-up visits by an oncology nurse or the patient's physician. Since all outcome measures are standard data recorded for these surgeries, no additional strain will be put on the participating patients. This study was granted approval by the institutional medical committee.

Data from the continuous monitoring of the anaesthesiologist are automatically saved in the patients’ medical file. These data will be used to identify the moments when the patient is in distress, that is, a sudden decrease of blood pressure, a sudden increase in heart rate, or a sudden decrease in oxygen saturation of the patient. These moments will be of special interest to the observers in order to observe the reaction of the team to sudden adverse events during surgery.

Data will be handled in a strictly confidential manner, and will be coded during the extraction of either patient characteristics or video analyses. It will be stored in a secure and encrypted database (research manager), and code lists will exclusively be stored at the hospital of consultation or treatment until video analysis results and patient characteristics have been matched. Afterwards, they will be destroyed. The video and audio data will be stored for a maximum period of 6 mo.

## Statistical analysis

Frequency statistics will be calculated for patient demographical data, and a Shapiro-Wilk test with *p* > 0.05 will be used to define normal distribution. Univariate analysis will be conducted to test for statistically significant differences in observation scores between open radical cystectomy and robot-assisted radical cystectomy cohorts across all variables, using independent sample *t* tests and Mann-Whitney *U* testing, as appropriate. A variable-selection strategy will be used to create multivariate models. Binary logistic regression will be conducted to calculate odds ratios and 95% confidence intervals for significant predictors on univariate analysis and clinically relevant covariates. Statistical significance is set at *p* < 0.05 based on a two-tailed comparison. Statistical analyses will be performed using SPSS Statistics version 24 (IBM, Armonk, New York, USA).

### Primary outcome measurements

The following outcomes will be reported.

Nontechnical skills will be observed using five different methods:

1. NOTSS [16]: The focus of the NOTSS assessment method lies on the following aspects of nontechnical skills:
  (a) Situation awareness: developing and maintaining dynamic awareness of the situation in operating theatre based on assembling of data from the environment, understanding of what they mean, and thinking ahead about what may happen next.
  (b) Decision making: skills for diagnosing the situation and reaching a conclusion in order to choose an appropriate course of action.
  (c) Communication and teamwork: skills for working in a team context to ensure that the team has an acceptable shared overview of the situation and can complete tasks effectively.
  (d) Leadership: leading the team and providing direction, demonstrating high standards of clinical practice and care, and being considerate about the needs of individual team members.
2. Oxford NOTECHS II [11], [12]: The focus of the NOTECHS II assessment method lies on the following aspects of nontechnical skills:
  (a) Leadership and management.
  (b) Teamwork and cooperation.
  (c) Problem solving and decision making.
  (d) Situation awareness.
3. OTAS [21]: ^The^ ^focus^ ^of^ ^the^ ^OTAS^ ^assessment^ ^method^ ^lies^ ^on^ ^the^ ^following^ ^aspects^ ^of^ ^nontechnical^ ^skills:^
  (a) Communication.
  (b) Coordination.
  (c) Cooperation and back-up behaviour.
  (d) Leadership.
  (e) Team monitoring and situational awareness.
4. ICARS [17]: The focus of the ICARS assessment method lies on the following aspects of nontechnical skills:
  (a) Checklist and equipment.
  (b) Interpersonal skills (communication and team skills, and leadership).
  (c) Cognitive skills (decision making and situational awareness).
  (d) Resource skills (stress and distractors).
  (e) Human factor analysis [22]: Human factor analysis consists of four levels of system failure: unsafe acts, preconditions for unsafe acts, unsafe supervision, and organisational influences.
5. Perioperative events (ie, people entering or leaving the OR, phone calls, etc.).

Technical skills in robot-assisted radical cystectomy will be analysed using two different methods:

1. GEARS [7]: The focus of the GEARS assessment method lies on general robot surgical principals, that is, depth perception, bimanual dexterity, efficiency, force sensitivity, autonomy, and robotic control.
2. GERT [5]: The focus of the GERT assessment method lies on the capture and analysis of technical errors and resulting events during laparoscopic procedures.

### Secondary outcome measurements

Age, World Health Organization performance status, Charlson comorbidity index, neoadjuvant chemotherapy, prior local treatment, prior radiation therapy in the surgical field, diagnosis, prior abdominal and/or pelvic surgery, indication of surgery, perioperative complications, postoperative complications according to the Clavien-Dindo system [24], length of hospital stay, stay in the intensive care unit, blood loss, patient-reported experience measures, patient-reported outcome measures, method of surgery, and oncological outcome (surgical margins and number of resected lymph nodes, and pathology results) will be registered prospectively. Patient follow-up will be for at least 30 d. Surgeon-specific data will be recorded (ie, age, gender, right or left handedness, gaming experience, and prior surgical and robot-assisted surgery experience of the surgeon).

### Regulation statement

As this is a prospective, observational, noninvasive study, participants will not be subject to any study treatments or actions. Even though the Medical Research Involving Human Subjects Act (in Dutch: Wet Medisch-wetenschappelijk Onderzoek met Mensen) does not imply that informed consent will be obtained, this study will be conducted in accordance to the “Code Goed Gebruik” (January 2002). Formal ethical approval has been provided by the Medical research Ethics Committees United (MEC-U), Nieuwegein (reference number W19.048). The study protocol is registered at the Netherlands Trial Registry under reference number NL8537.

### Privacy

Observations during surgery will be performed by two members of the urology in-house staff (medically trained researchers with training in the analysis of both nontechnical and technical skills); none of the observers have a hierarchical relationship with any of the team members.

As discussion of planned surgeries is part of daily staff meetings, there are no additional privacy concerns.

The observations do not contain the name of the patient, or the date and time of surgery. This is in accordance with the General Data Protection Regulation.

### Handling and storage of data and documents

Data will be handled in a strictly confidential manner, and will be coded during the extraction of patient characteristics and video analysis. It will be stored in a secure and encrypted database (research manager), and code lists will exclusively be stored at the hospital of consultation or treatment until video analysis results and patient characteristics have been matched. The data will be stored for a maximum period of 6 mo. Afterwards, they will be destroyed.

***Author contributions*****:** Alexander J.W. Beulens had full access to all the data in the study and takes responsibility for the integrity of the data and the accuracy of the data analysis.

*Study concept and design:* Wagner, Beulens.

*Acquisition of data*: Beulens, Brinkman.

*Analysis and interpretation of data*: Beulens.

*Drafting of the manuscript*: Beulens, Brinkman.

*Critical revision of the manuscript for important intellectual content:* Hendrikx, Bangma, van Merriënboer, Koldewijn, van Basten, Wagner, van der Poel, Brinkman, Beulens

*Statistical analysis*: Beulens.

*Obtaining funding:* Hendrikx, Koldewijn, Brinkman, Wagner, van der Poel.

*Administrative, technical, or material support:* Brinkman, Koldewijn, Beulens.

*Supervision:* Brinkman, Wagner, van der Poel, Bangma.

*Other:* None.

***Financial disclosures:*** Alexander J.W. Beulens certifies that all conflicts of interest, including specific financial interests and relationships and affiliations relevant to the subject matter or materials discussed in the manuscript (eg, employment/affiliation, grants or funding, consultancies, honoraria, stock ownership or options, expert testimony, royalties, or patents filed, received, or pending), are the following: None.

*Funding/Support and role of the sponsor*: This work was supported by funding from Astellas Pharma Europe Ltd. and Olympus Netherlands B.V.

## References

[1] Dutch Health Inspectorate (Inspectie voor de Gezondheidszorg). (2010). Insufficient carefulness at the introduction of surgical robots [Onvoldoende zorgvuldigheid bij introductie van operatierobots]..

[2] Porte P.J., Verweij L.M., Bekkers R.L.M. (2017). Robotic surgery for medical specialists, basic proficiency requirements for the safe use of robotic surgery.

[3] Brinkman W., de Angst I., Schreuder H. (2017). Current training on the basics of robotic surgery in the Netherlands: time for a multidisciplinary approach?. Surg Endosc Other Interv Tech.

[4] Birkmeyer J.D., Finks J.F., O’Reilly A. (2013). Surgical skill and complication rates after bariatric surgery for the Michigan Bariatric Surgery Collaborative. N Engl J Med.

[5] Husslein H., Shirreff L., Shore E.M., Lefebvre G.G., Grantcharov T.P. (2015). The generic error rating tool: a novel approach to assessment of performance and surgical education in gynecologic laparoscopy. J Surg Educ.

[6] Goldenberg M.G., Lee J.Y., Kwong J.C.C., Grantcharov T.P., Costello A. (2018). Implementing assessments of robot-assisted technical skill in urological education: a systematic review and synthesis of the validity evidence. BJU Int.

[7] Goh A.C., Goldfarb D.W., Sander J.C., Miles B.J., Dunkin B.J. (2012). Global evaluative assessment of robotic skills: Validation of a clinical assessment tool to measure robotic surgical skills. J Urol.

[8] Hussein A.A., Ghani K.R., Peabody J. (2017). Development and validation of an objective scoring tool for robot-assisted radical prostatectomy: prostatectomy assessment and competency evaluation. J Urol.

[9] Beulens A.J.W., Brinkman W.M., Van der Poel H.G. (2019). Linking surgical skills to postoperative outcomes: a Delphi study on the robot-assisted radical prostatectomy. J Robot Surg.

[10] Goldenberg M.G., Goldenberg L., Grantcharov T.P. (2017). Surgeon performance predicts early continence after robot-assisted radical prostatectomy. J Endourol.

[11] Robertson E.R., Hadi M., Morgan L.J., Roma P.G. (2014). Oxford NOTECHS II: a modified theatre team non-technical skills scoring system.

[12] Mishra A., Catchpole K., McCulloch P. (2009). The Oxford NOTECHS system: reliability and validity of a tool for measuring teamwork behaviour in the operating theatre. Qual Saf Health Care.

[13] Moorthy K., Munz Y., Adams S., Pandey V., Darzi A. (2005). A human factors analysis of technical and team skills among surgical trainees during procedural simulations in a simulated operating theatre. Ann Surg.

[14] Flin R., Patey R. (2011). Non-technical skills for anaesthetists: developing and applying ANTS. Best Pract Res Clin Anaesthesiol.

[15] Mitchell L., Flin R., Yule S., Mitchell J., Coutts K., Youngson G. (2013). Development of a behavioural marker system for scrub practitioners’ non-technical skills (SPLINTS system). J Eval Clin Pract.

[16] Sharma B., Mishra A., Aggarwal R., Grantcharov T.P. (2011). Non-technical skills assessment in surgery. Surg Oncol.

[17] Raison N., Wood T., Brunckhorst O. (2017). Development and validation of a tool for non-technical skills evaluation in robotic surgery—the ICARS system. Surg Endosc.

[18] Gjeraa K., Spanager L., Konge L., Petersen R.H., Østergaard D. (2016). Non-technical skills in minimally invasive surgery teams: a systematic review. Surg Endosc.

[19] van der Vliet W.J., Haenen S.M., Solis-Velasco M. (2019). Systematic review of team performance in minimally invasive abdominal surgery. BJS Open.

[20] McBride K.E., Steffens D., Duncan K., Bannon P.G., Solomon J. (2019). Knowledge and attitudes of theatre staff prior to the implementation of robotic-assisted surgery in the public sector. PLoS One.

[21] Undre S., Sevdalis N., Healey A.N., Darzi A., Vincent C.A. (2007). Observational teamwork assessment for surgery (OTAS): refinement and application in urological surgery. World J Surg.

[22] Patidar N., Yadav P., Sureka S.K., Mittal V., Kapoor R., Mandhani A. (2016). An audit of early complications of radical cystectomy using Clavien-Dindo classification. Indian J Urol.

[23] The Royal College of Surgeons of Edinburgh (2019). Non-technical skills for surgeons (NOTSS)..

[24] Cohen T.N., Wiegmann D.A., Reeves S.T., Boquet A.J., Shappell S.A. (2017). Coding human factors observations in surgery. Am J Med Qual.

