# Supplementary material for: A Prospective, Observational, Multicentre Study Concerning Nontechnical Skills in Robot-assisted Radical Cystectomy Versus Open Radical Cystectomy
Source: Eur Urol Open Sci. 2020 Jul 3;19:37–44. doi: 10.1016/j.euros.2020.05.003 (PMC8317860; doi:10.1016/j.euros.2020.05.003)
Supplement: Supplementary file 1 [file mmc1.docx]

**Supplementary material**

Survey on potential benefits of Robot assisted surgery developed by McBride et al [1].

**Based on your current understanding of robotic surgery, please rate your level of agreement with the following statements**

| **6. Benefits of Robot Assisted Surgery for patients:** | **Disagree** | **Neutral** | **Agree** |
| --- | --- | --- | --- |
| Robotic surgery will help reduce overall length of stay | ⃝ | ⃝ | ⃝ |
| Robotic surgery will reduce patient post-operative pain | ⃝ | ⃝ | ⃝ |
| Robotic surgery will reduce intraoperative complications (e.g. blood loss) compared to current procedures | ⃝ | ⃝ | ⃝ |

| **7. Benefits of Robot Assisted Surgery for staff:** | **Disagree** | **Neutral** | **Agree** |
| --- | --- | --- | --- |
| Robotic surgery increases the value of staff roles | ⃝ | ⃝ | ⃝ |
| Robotic surgery will increase my job satisfaction | ⃝ | ⃝ | ⃝ |
| Being involved in robotic surgery enhances overall staff knowledge | ⃝ | ⃝ | ⃝ |

| **8. Benefits of RAS for workplace Environment:** | **Disagree** | **Neutral** | **Agree** |
| --- | --- | --- | --- |
| Robotic surgery is a Work Health and Safety (WH&S) concern (additional equipment in the operating theatre) | ⃝ | ⃝ | ⃝ |
| Care and handling of specialised robotic surgery equipment concerns me | ⃝ | ⃝ | ⃝ |
| I am concerned about maintenance of sterile field when assisting in robotic surgery cases more so than when assisting in other cases | ⃝ | ⃝ | ⃝ |
| I am concerned about a decrease in my direct involvement in the case intraoperatively during robotic surgery compared to other procedures | ⃝ | ⃝ | ⃝ |
| I am concerned that robotic surgery will increase operating time | ⃝ | ⃝ | ⃝ |
| I am concerned regarding space and location of the robot | ⃝ | ⃝ | ⃝ |
| I am concerned that robotic surgery will negatively affect current team dynamics in the operating theatre | ⃝ | ⃝ | ⃝ |
| I am concerned that robotic surgery will add significant cost and  financial pressure on our facility | ⃝ | ⃝ | ⃝ |

| **9. Facilitators towards the implementation of new**  **technology:** | **Disagree** | **Neutral** | **Agree** |
| --- | --- | --- | --- |
| Formal, theoretical training | ⃝ | ⃝ | ⃝ |
| Practical training (i.e. simulation) | ⃝ | ⃝ | ⃝ |
| Educational guides and references for use intraoperatively | ⃝ | ⃝ | ⃝ |
| Support staff available when required | ⃝ | ⃝ | ⃝ |

**References**

[1] McBride KE, Steffens D, Duncan K, Bannon PG, Solomon J. Knowledge and attitudes of theatre staff prior to the implementation of robotic-assisted surgery in the public sector. PLoS One. 2019;14(3):1–8.
